# Research priorities for the sustainability of coral-rich western Pacific seascapes

**DOI:** 10.1007/s10113-023-02051-0

**Published:** 2023-04-21

**Authors:** Graeme S. Cumming, Maja Adamska, Michele L. Barnes, Jon Barnett, David R. Bellwood, Joshua E. Cinner, Philippa J. Cohen, Jennifer M. Donelson, Katharina Fabricius, R. Quentin Grafton, Alana Grech, Georgina G. Gurney, Ove Hoegh-Guldberg, Andrew S. Hoey, Mia O. Hoogenboom, Jacqueline Lau, Catherine E. Lovelock, Ryan Lowe, David J. Miller, Tiffany H. Morrison, Peter J. Mumby, Martin Nakata, John M. Pandolfi, Garry D. Peterson, Morgan S. Pratchett, Timothy Ravasi, Cynthia Riginos, Jodie L. Rummer, Britta Schaffelke, Thomas Wernberg, Shaun K. Wilson

**Affiliations:** 1 Australian Research Council Centre of Excellence for Coral Reef Studies, James Cook University, Townsville, QLD 4811 Australia; 2 Australian Research Council Centre of Excellence for Coral Reef Studies, Australian National University, Canberra, Australia; 3 Research School of Biology, Australian National University, Canberra, Australia; 4 School of Geography, Earth, and Atmospheric Sciences, University of Melbourne, Melbourne, Australia; 5 College of Science and Engineering, James Cook University, Townsville, Australia; 6 WorldFish, Penang, Malaysia; 7 Australian Institute of Marine Science, Townsville, Australia; 8 Crawford School of Public Policy, Australian National University, Canberra, Australia; 9 ARC Centre of Excellence for Coral Reef Studies, The University of Queensland, Brisbane, Australia; 10 School of Biological Sciences, The University of Queensland, Brisbane, Australia; 11 Australian Research Council Centre of Excellence for Coral Reef Studies, University of Western Australia, Perth, Australia; 12 Oceans Institute, University of Western Australia, Perth, Australia; 13 College of Public Health, Medical & Veterinary Sciences, James Cook University, Townsville, 4811 Australia; 14 Indigenous Education and Research Centre, James Cook University, Townsville, 4811 Australia; 15 Stockholm Resilience Centre, Stockholm University, Stockholm, Sweden; 16 Marine Climate Change Unit, Okinawa Institute of Science and Technology (OIST), 1919-1 Tancha, Onna-Son, Okinawa Japan; 17 Institute of Marine Research, Floedevigen Research Station, Nis, Norway; 18 Western Australia Government Department of Biodiversity, Conservation and Attractions, Perth, Australia

**Keywords:** Marine, Ocean, Resilience, Social-ecological, Development, SDG, Coral reef

## Abstract

Nearly a billion people depend on tropical seascapes. The need to ensure sustainable use of these vital areas is recognised, as one of 17 policy commitments made by world leaders, in Sustainable Development Goal (SDG) 14 (‘Life below Water’) of the United Nations. SDG 14 seeks to secure marine sustainability by 2030. In a time of increasing social-ecological unpredictability and risk, scientists and policymakers working towards SDG 14 in the Asia–Pacific region need to know: (1) How are seascapes changing? (2) What can global society do about these changes? and (3) How can science and society together achieve sustainable seascape futures? Through a horizon scan, we identified nine emerging research priorities that clarify potential research contributions to marine sustainability in locations with high coral reef abundance. They include research on seascape geological and biological evolution and adaptation; elucidating drivers and mechanisms of change; understanding how seascape functions and services are produced, and how people depend on them; costs, benefits, and trade-offs to people in changing seascapes; improving seascape technologies and practices; learning to govern and manage seascapes for all; sustainable use, justice, and human well-being; bridging communities and epistemologies for innovative, equitable, and scale-crossing solutions; and informing resilient seascape futures through modelling and synthesis. Researchers can contribute to the sustainability of tropical seascapes by co-developing transdisciplinary understandings of people and ecosystems, emphasising the importance of equity and justice, and improving knowledge of key cross-scale and cross-level processes, feedbacks, and thresholds.

## Introduction

The well-being, prosperity, and security of over nearly a billion coastal people living in 117 countries are already at risk from degradation in marine and coastal environments (Sing Wong et al. 2022). Projections suggest that unless environmental instability and social inequity in seascapes are addressed, more than 1.3 billion people will be at risk by 2050 (Lam et al. 2020). As the marine environment is degraded, declines in regulatory services will increase human exposure to storms, flooding, and pathogens; and the supply of key provisioning and cultural ecosystem services, such as food production and tourism, will become lower and less reliable (Karki et al. 2018; Sing Wong et al. 2022).

In 2015, world leaders under the United Nations agreed on a set of 17 global commitments to secure a sustainable future for humanity. Among these Sustainable Development Goals (SDGs), SDG 14 (‘Life below water’) seeks explicitly to ‘conserve and sustainably use the oceans, seas, and marine resources for sustainable development’. Although key milestones were intended to be achieved by 2030, many targets of SDG 14 appear unlikely to be met (Jouffray et al. 2020). The gap between reality and SDG 14 is particularly pronounced in the tropics, where accelerating ecological degradation and increasing global demand for marine resources are placing increasingly more people at risk and the condition of marine ecosystems appears to be diverging rather than converging with the objectives of SDG 14.

**Fig. 1:**
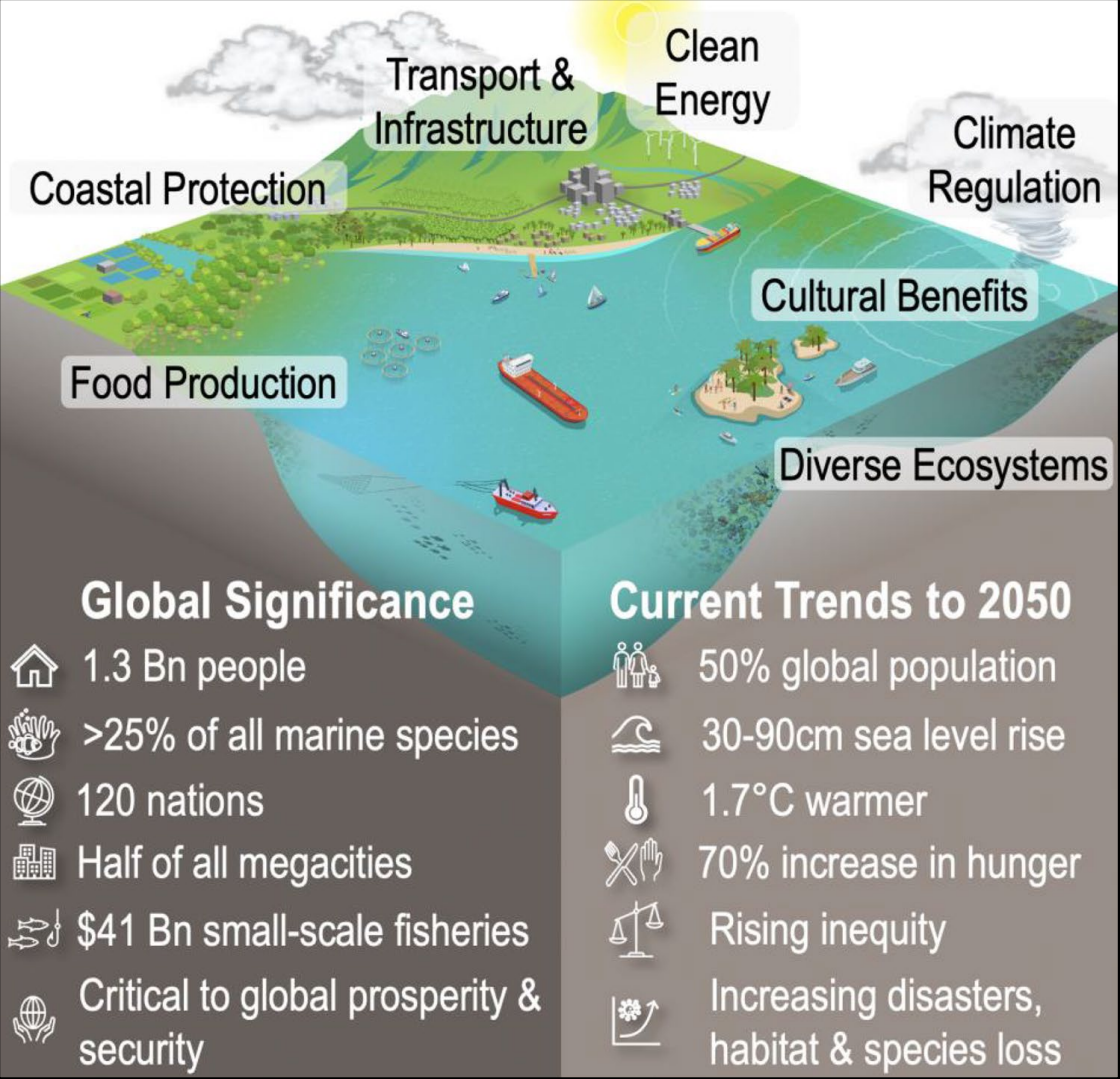
Overview of some of the key elements, significance, and trends for tropical seascapes. (Figure credit: Jerker Lokrantz)

In this paper, we consider how research can contribute towards achieving SDG 14, particularly in relation to the tropical, coral-rich marine seascapes in the western Asia–Pacific region where we live and work. We define a seascape as a geographically bounded, heterogeneous space encompassing coastal and marine ecosystems and societies (Fig. 1; Pittman (2017); Turner et al. (2001)). As ecological, socioeconomic, and political units, seascapes typically incorporate multiple interconnected uses, sectors, property rights systems, and governance mechanisms in addition to their biophysical and ecological dynamics. The presence of social-ecological feedbacks and human-moderated tradeoffs between different ecosystem services create a series of complex interactions between different seascape elements, making it challenging to predict their dynamics and to develop robust policy for sustainable development.

In trying to make progress towards SDG 14, we identified three overarching questions as being critical for framing future research: (1) How and why are seascapes changing? (2) What can and should society do about these changes? and (3) How can science and society together achieve sustainable seascape futures? While these questions are applicable to many ecosystems and landscapes around the world, they are particularly important for tropical seascapes, which are less documented and less researched than most temperate marine or terrestrial systems (Bernard et al. 2021; Feeley et al. 2017; Menegotto and Rangel 2018). Drivers of change in tropical marine ecosystems are often poorly established; human impacts are harder to demonstrate and there is less baseline data; and marine tenure and ownership (and hence, responsibility) may be harder to demarcate. Achieving the goals of SGD14 will be impossible without understanding what ‘conserve and sustainably use’ means for fast-changing marine ecosystems and embedding or co-producing this understanding in a context of traditional knowledge and local needs. Broad-scale biodiversity conservation, for example, demands the reduction or elimination of harmful influences, the protection of areas and networks of ecologically functional habitat, and the restoration of degraded areas (Fischer et al. 2006; Poiani et al. 2000). These activities depend on understanding both the drivers of ecosystem change and the ways in which societies can successfully intervene to influence them. Achieving SDG14 will only be possible through the adoption of deliberate management actions; and consistent with adaptive management principles, these should ideally be iteratively guided, monitored, and improved through social-ecological research (e.g. Gormley et al. 2015; Nickols et al. 2019). Thus, in this article, we present a vision for a series of interlinked research programmes, embedded in societies, that can directly interface with social, economic, and political elements of Asia–Pacific seascapes through a series of interactive feedbacks between science, policy, and practice.

*Challenge 1: How and why are tropical seascapes changing?* The increasing rate and magnitude of climate change, coastal development, land use change, and marine exploitation are in combination causing escalating, unpredictable, and often undesirable changes in tropical seascapes (Hughes et al. 2017a; Murray et al. 2019). For instance, the capacity of coral reefs to provide ecosystem services has declined by half since the 1950s (Eddy et al. 2021). Loss of seagrass meadows, driven by coastal development and poor water quality, has been widespread and substantial (Dunic et al. 2021); land-use change has led to the loss of 20–35% of global mangrove extent since 1970 (Friess et al. 2019); and a large majority of global coastal and marine fish stock are now fully exploited, overexploited, or depleted (FAO 2020). Nutrient discharges from the land remain problematic in many areas despite global efforts to reduce eutrophication and benthic hypoxia (Duarte and Krause-Jensen 2018).

The cumulative effects of multiple stressors on key processes that link individual ecosystems are unclear; and the influence of seascape change on fundamental longer-term biological processes, such as evolution and extinction, threatens the persistence of marine biodiversity. Moreover, as some habitats benefit at the expense of others, new processes and feedbacks are altering the provision of ecosystem goods and services and providing new opportunities and risks for society. For instance, regime shifts from coral to macroalgal dominated reefs diminish ecosystem service provision by corals and pose risks to human health, but may enhance blue carbon storage and benefit some fisheries (Hill et al. 2015; Wilson et al. 2022). More and better data, and an improved understanding of system dynamics, are needed about the nature and causes of seascape change before these different trends and interactions can be managed to achieve the sustainable conservation and use targets of SDG 14.

*Challenge 2: What can and should society do about seascape change?* Increasingly novel and unpredictable feedbacks between ecosystems and society are developing. Seascapes in the Western Pacific contain dynamic and complex interactions among organisms including humans, systems, and places, across diverse scales (Pittman et al. 2021; Saunders et al. 2014). Key interconnections include, for example, how ‘telecoupling’ links distant places through globalisation, supply chains, and markets (Eakin et al. 2014), how changes in species composition affect human health in direct and indirect ways, and how different political jurisdictions interact to resolve the challenges of seascape management (Spijkers et al. 2018).

Governmental policies and actions are often inadequate to address SDG 14. In many countries, land and sea are governed under separate agencies, and conservation and exploitation of the same ecosystem are separated (e.g. in Australia, marine parks and fisheries management are in different governmental departments). Different policies may be poorly aligned or in conflict with one another and with SDG 14, as in the case of guidelines for small-scale fisheries within the EU (e.g. Said and Chuenpagdee (2019); noting also that EU policy and law directly affect many island territories in the Pacific, such as French Polynesia and New Caledonia). Coordination of policy and management at appropriate scales is another importance governance challenge. For example, in the Philippines, efforts to coordinate localised seascape management by ‘Local Governmental Units’ through broader-scale ‘Bay Management Councils’ had to be abandoned due to self-interest and a lack of cooperation (Pomeroy et al. 2010). SDG 14-related solutions proposed by governments to meet the needs of particular beneficiaries, such as tourism operators or commercial fishers, often ignore tradeoffs, feedbacks, and synergies among sectors and across scales (Charles 2012); and policy intended to increase the delivery of one ecosystem service can undercut the provision of others (Hodgson et al. 2019; Liu et al. 2015). For example, food provisioning involves removing fish from the sea, but reducing the abundance of fish can impact other services they provide (e.g. the regulation of algal growth on corals).

*Challenge 3: How can science and society together achieve sustainable seascape futures?* Deficiencies and injustices that result in reduced social-ecological sustainability can arise from past or present marine governance (Kelly et al. 2019; Nunan et al. 2020). Marine governance systems frequently marginalise specific resource users and exacerbate inequalities, which can create unjust and unsustainable outcomes and undermine the legitimacy and efficacy of policy (Engler 2020), impeding efforts towards SDG14. However, new approaches to governance that delegate management authority and decision-making to local communities or embrace new technologies (e.g. reef restoration using genetically modified corals that are heat-resistant) can also displace some resource users and create winners and losers (Bennett et al. 2021; Cinner et al. 2014). Developing approaches to marine governance that fairly and efficiently address problems, while coping with rapid change and external pressures (Morrison et al. 2020), will be key for SDG 14. By adopting a more action-focused, socially aware agenda and integrating more deliberately into the wider community of policy and practice, we argue that researchers can become agents of positive change rather than merely observers of social or ecological declines.

### Addressing the challenges

Each of these three challenges to achieving SDG 14 has both independent and inter-dependent aspects. Marine and coastal changes are embedded in feedback loops of anthropogenic action; climate change, overfishing, and deforestation, for example, can be seen as both cause and consequence of governance and market failures. As cascading effects ramify through social-ecological systems, they create their own dynamics that present new obstacles to effective, sustainable, and equitable governance (Rocha et al. 2018; Song et al. 2018). Thus, addressing all three challenges simultaneously and in an integrated manner is critical. Achieving SGD 14 will require revised research and policy priorities, supported by novel processes of impact-focused knowledge generation and broad consensus and collaboration across funding agencies and the research community. Research that advances progress towards SDG 14 must not only combine understandings of ecological and socioeconomic change, but also link different approaches for collecting, analysing, and integrating knowledge and data (Opdam et al. 2018). This article addresses the first piece of this problem, the question of how to focus research and enhance its contributions to social-ecological outcomes, presenting the results of a horizon scan which seeks to highlight key research priorities to help navigate the three challenges and support SDG 14. We do not seek to directly make policy recommendations in this article; our focus is deliberately on the role of researchers and the ways in which improved integration of research into practice and policy can produce improved outcomes.

## Methodology and participants

We conducted a series of horizon scanning workshops run under the auspices of the ARC Centre of Excellence in Coral Reef Studies, a global consortium of universities and partners in tropical coastal and marine science and practice. We used a group collaborative process of discussion and debate to explore and synthesise our views on the future of tropical marine science, and identify research priorities. Through the process we followed the three broad aims of horizon scanning (Könnölä et al. 2012; Sutherland and Woodroof 2009) to (1) articulate credible observations about current or imminent changes in marine social-ecological systems; (2) identify new and emerging issues that may have received insufficient attention; and (3) develop ideas that could be meaningfully shared, elaborated, and discussed by the participants. Unlike more typical horizon scans, which seek to identify emerging perturbations, the agenda of our workshops was specifically focused on research and its role in the future of reef-dominated seascapes.

Participants included a diverse, gender-balanced range of leading scientists covering a wide range of marine science expertise (e.g. including experts in social, economic, ecological, and evolutionary fields) and career stages. While the majority of participants were based at Australian institutions due to COVID-19 restrictions and the preference that we meet in person, the group was considerably more diverse than institutional affiliations would suggest; it included individuals of seven different nationalities with extensive experience working in locations beyond Australia (e.g. Fiji, Indonesia, Japan, Malaysia, Papua New Guinea, Solomon Islands, East Africa, South Africa), with Indigenous communities, and with societal partners (e.g. the Great Barrier Reef Marine Park Authority, various protected area agencies, and WorldFish).

Our initial scoping process identified the three challenges discussed above as being of direct relevance to both national and local decision-makers (e.g. policy makers, governmental agencies, urban planners, protected area managers). After a series of smaller-group discussions, we collated suggestions and reduced our list of candidate research priorities to the nine summarised in this manuscript through a process of debate and discussion. Although identifying specific pathways to achieve desirable outcomes was beyond the scope of this manuscript, we compared our findings to future-oriented global frameworks (i.e. international science policy processes, many of which map out pathways to achieving their goals). This was done after, rather than before or during, the process to maintain a level of creativity and independence from existing paradigms during the process and to evaluate external validity after the process.

## Research priorities

**Fig. 2:**
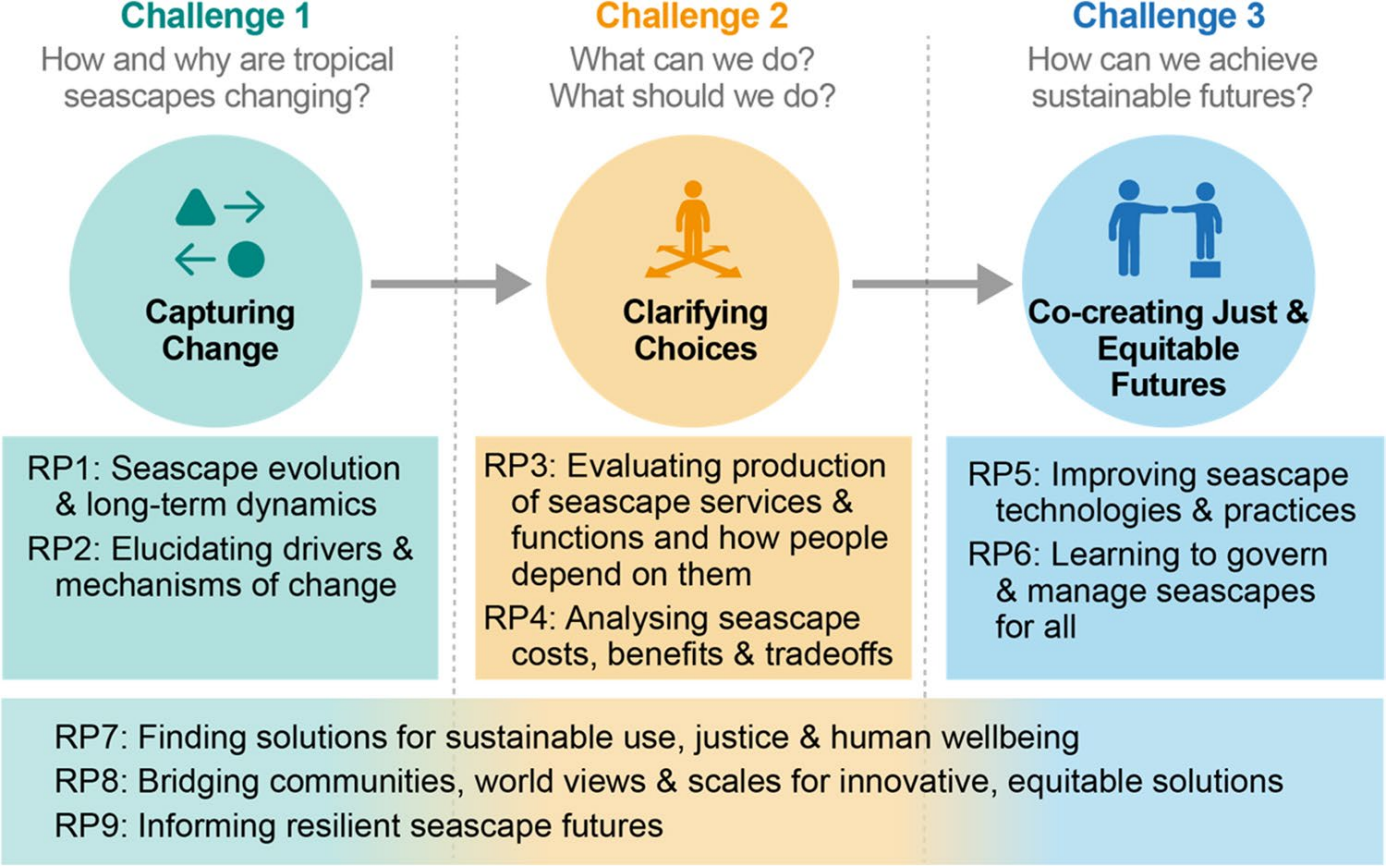
Summary of the nine identified research priorities. This figure shows how each of the priorities contributes to addressing the three challenges for the sustainability of tropical seascapes. RP stands for ‘Research Priority’

Realising the full potential of research and adaptive management in fostering and co-creating sustainable seascape futures will require a cohesive approach that addresses all three challenges. We identified nine distinct but interlocking research priorities (RPs) that capture what we perceive to be the primary ingredients of the solution. These priorities explicitly consider strategies for navigating the complexity of geographic connections, feedbacks between cause and effect, and cross-scale processes (Fig. 2 and Table 1). They span the full range of relevant disciplines and should in theory produce knowledge at each level (or within each discipline) that is critical for addressing questions at other levels and through other disciplines. For example, an understanding of how fishing impacts fish communities (RP2) is critical for projecting food security (RP3) and clarifying the tradeoffs between other industries and fisheries (RP4); these in turn can be better regulated and modified by governance and management if they are well understood (RP6), and should provide greater and more lasting benefits to people if they are designed to be sustainable and just (RP7).

**Table 1:** Summary of the nine critical research priorities, the challenges and focal questions they address, the minimum set of kind(s) of co-production they require, and the contributions they can make to enhancing global seascape sustainability. Three research priorities are universal to all three critical challenges; the other six priorities are more specific, but all are interconnected. Note that this table identifies research needs but does not attempt to define pathways to achieving these outcomes (which would require a larger and more inclusive exercise)

Research priority	Challenge	Examples of focal questions	Anticipated research contribution
1. Seascape geological and biological evolution and adaptation	1	• Where, when, and how fast have species adapted to past changes? • How do within-lifetime, cross-generational, and historical environmental fluctuations and extremes continue to shape biological diversity and biogeography? • How, where, and how fast have ecological communities changed and what are the consequences for ecological function?	Develop an improved understanding of capacity for biological adaptation and the evolution of species interactions and interdependencies in seascapes. This understanding will inform analyses of future changes in the geological and biological components of seascapes as ocean conditions change
2. Elucidating drivers and mechanisms of change	1	• How will environmental extremes and perturbations (e.g. climate variability and change, acidification, cyclones, sea level rise, pollution) influence seascapes? • How will simultaneous change in inter-connected ecosystems within the same seascape (e.g. corals, seagrass, mangroves, algae) interact with broader-scale socioeconomic trends affecting human societies (e.g. changing markets, new technologies, global politics)? • What factors at different scales drive or underpin human responses to seascape change? How have humans societies co-developed with seascapes?	Explore both proximate and local drivers of change, including critical wider societal and economic influences
3. How are seascape functions and services produced, and how do people depend on them?	2	• How do differences in species composition lead to differences in ecosystem-level services (e.g. food production, coastal protection, water quality) to people? • How do biophysical heterogeneity and societal diversity create differences and tradeoffs in seascape benefits received by people? • How do seascape functions and benefits to people change locally and regionally under future scenarios?	Understand the feedbacks within and between seascape components and the ways in which seascape changes affect different societal groups, including those who live far from seascape ecosystems but still have an interest in them
4. Costs, benefits and trade-offs to people in changing seascapes	2	• What alternate visions exist of just and sustainable novel land-sea economies, and how and why do they differ among groups? • What enablers or barriers shape the possible responses of seascape-dependent societies? • How can social-ecological transitions (e.g. from unsustainable to sustainable practices) arise through existing processes, governance structures, or innovations under current conditions?	Anticipate and draw together alternate understandings and visions of transitions
5. Improving seascape technologies and practices	3	• Which technologies and practices can enable seascapes to respond effectively to change? • How do costs and benefits of nature-based solutions vary spatially and for different societal groups? • How and where do synergies arise between strategies for enhancing rural and regional development?	Determine key factors (biophysical, social, economic, governance) affecting the sustainability of seascapes and seascape-dependent societies in the face of emerging threats such as ocean warming, sea level rise, and impacts of infectious disease
6. Learning to govern and manage seascapes for all	3	• What different policy and institutional solutions exist, and for whom? • How do different solutions manage perceptions, risk, feasibility, and ethics? • How are sustainable marine futures enabled through best-practice policy and governance of changing marine ecosystems?	Resolve key basic knowledge gaps (externalities, distal drivers, unidentified global and macro-economic, geo-political and health risks) and practical challenges (engagement, equity, legitimacy, and efficacy) with important lessons for policy and management
7. Sustainable use, justice, and human well-being	1, 2, 3 (cross-cutting)	• How do seascape-dependent societies prioritise risks and assess plausible futures? • What are the benefits and trade-offs associated with different responses to sustaining seascapes, and how are they distributed within and across social groups?	Advance knowledge of how environmental and social drivers in seascapes (e.g. changes in mangrove or seagrass habitats (Friess et al. 2019)) affect human well-being, including food security, cultural identity, livelihoods, and physical and mental health
8. Bridging communities and epistemologies for innovative, equitable, and scale-crossing solutions	1, 2, 3 (cross-cutting)	• How do different ontologies (explanations of the world) and epistemologies (ways of knowing) underpin approaches to seascapes and understandings of the future? • To what extent are the differences complementary, and how can we re-frame scientific understanding (which has largely ignored traditional knowledge) for further knowledge generation?	Research co-produced at the interface between Western scientific knowledge systems and First Nations knowledge systems will create new perspectives on environmental stewardship; facilitate taking local solutions global; and expand the solution space for achieving a just and sustainable future for seascapes
9. Informing resilient seascape futures	1, 2, 3 (cross-cutting)	• Where and when do tipping points, feedbacks, synergies, and cascading effects (e.g. impacts of coral reef state shifts on food security for island communities) occur in seascapes? • How will emerging global trends influence the resilience of social-ecological states in seascapes?	New methods for evaluating and simulating, from diverse perspectives, transformation and fundamental systemic change; cross-scale approaches that bridge from molecules to global politics (e.g. linking the adaptive capacity of coral symbionts via sustainability of fisheries to action limiting CO_2_ emissions); and a diverse ensemble of social-ecological models that can be used to explore potential implications and impacts of relevant changes at different scales

Addressing the first challenge (changing seascapes) will require new understandings of seascape dynamics and continual updates of knowledge. We identified two key research priorities that help address this: seascape evolution and adaptation (RP1), and elucidating drivers and mechanisms of change (RP2; Table 1). Instead of merely sharing reports of individually degraded coastal ecosystems (Dunic et al. 2021; Friess et al. 2019; Hughes et al. 2017b; Murray et al. 2019), a coherent approach is needed to interpret the meaning of ongoing social and ecological changes for human well-being (Box 1) and ecological processes across diverse scales. Coherence emerges from both short- and long-term analyses of marine social-ecological change, requiring a synthesis of evolutionary, ecological, and societal perspectives. Given the immediacy of environmental change, rapid evolutionary responses and their interactions with human influences are a particular research priority. Open questions remain about the causes and relevance of shorter-term variation in organismal physical structure, physiology or behaviour that is not due to the genome, the degree to which this variation can be transmitted between generations, and the implications of short-term responses for population genetics (Donelson et al. 2019). Successful adaptation is supported by larger population sizes; but trade-offs between relevant traits (e.g. environmental tolerance vs. growth rate) and spatial variation in the environment may limit adaptation (Coleman and Wernberg 2020; Walsh and Blows 2009). Similarly, many details of marine dispersal and connectivity and their impact on evolutionary processes remain unclear (Sanford and Kelly 2011); and little is known about how biotic interactions will further compound the complexity of these processes. For example, rapid ecological adaptation may be enabled by interspecific hybridisation (Oziolor et al. 2019) or hindered by emerging marine pathogens (Burge et al. 2014).

**Box 1** Human wellbeing

**Table:** 

Seascape condition shapes human well-being in diverse ways, including material processes that influence work and income from industries such as fishing and tourism; affective and cognitive responses to changes in important places, species, and landscapes; and changes in the composition and roles of local human communities, policies, and strategies (Gibson et al. 2019). Well-being can be understood broadly as living a life that is comfortable, secure, healthy, and happy. In addition to its material dimensions, well-being is also a function of subjective psycho-physiological processes and social relations, which are in turn influenced by individual characteristics (e.g. age, class, culture, gender, and livelihood) and social-ecological location and context (Warr 2012). Understanding human well-being is critical for achieving ecological sustainability because well-being is a dominant influence on decisions about how to use and manage seascapes, it influences self-efficacy, and it is a desired outcome of effective governance	

Analysis of tropical marine ecosystems and people has often concentrated on a few locations that do not always correspond to the distribution of ecological values and threats (Fisher et al. 2011; Partelow et al. 2018). However, if scientific findings are to help societies to anticipate and respond more proactively to new or emerging environmental problems (Hughes et al. 2005; Liu et al. 2007), research will need to consider both remote and heavily peopled seascapes and explain how increasing ecological change affects human values and well-being: e.g. are there feedbacks between changes in marine habitat structure and attitudes to resource use? What are the socioeconomic implications of the potential degradation of small-scale fisheries for users of other resources in impacted seascapes? How do demographic trends influence the use of marine ecosystems? For example, Lapointe et al. (2020) found that urbanisation in the Solomon Islands affected people’s ecosystem services preferences, changing the nature of their interaction with marine ecosystems. Disturbance regimes in the West Pacific are changing, and the loss and fragmentation of habitats is altering the connectivity and composition of both habitat-forming and habitat-associated taxa (Saunders et al. 2014). Integrative research is particularly needed to extend and connect understandings of the different pieces of chains of causality and make their broader relevance to people and broad-scale sustainability concerns explicit. For example, while we know that shifts in marine habitats will impact fish populations, the magnitude and relative importance of these impacts for the livelihoods and nutritional status of seascape populations remain unclear (Mellin et al. 2022). Efforts under SDG-14 to conserve and sustainably use marine resources, such as the creation of new protected areas or imposing new restrictions on fisheries, will have to take into account the changing, dynamic nature of both social-ecological relationships and the perturbations they experience (Cumming et al. 2005).

Addressing the second challenge (what can and should society do) requires clarifying the complex interactions among different system components, including, for example, cross-scale interactions, feedbacks, knock-on effects, and interacting thresholds (Cumming et al. 2017). We identified two key research priorities: understanding how seascape functions and services are produced, and how people depend on them (RP3); and costs, benefits, and trade-offs to people in changing seascapes (RP4; Table 1). Current scientific understanding cannot definitively state how changes in marine habitats (e.g. seagrass, mangroves, coral reefs) and their connections will influence broad-scale patterns in the abundance and ecological functions of marine organisms. Analysis of ecosystem functions in many marine and coastal systems has been held back by an inability to directly measure the storage or movement of energy or material (Bellwood et al. 2019). Several recent community-level estimates of material fluxes across ecosystem boundaries reveal an extensive contribution of spatial subsidies to fish productivity (Graham et al. 2018; Morais and Bellwood 2019), but the generality of these results is unclear and improvements in both methods and underlying theory for exploring material fluxes are needed. Conversely, threats such as terrestrial sediments and climate change may impede marine ecosystem functions; preliminary studies offer surprising insights and suggest new areas for research. For example, a collapse of both algal and fish production on coral reefs can follow chronic sediment increases (Tebbett et al. 2021), while extensive coral loss may have unexpectedly small effects on fish productivity (Morais et al. 2020) and fisheries (Robinson et al. 2019).

Changes in coastal and marine ecosystems are expected to have a profound influence on human societies, but understanding of how societies might respond is still rudimentary (Barnes et al. 2020). Seascape ecosystem services provide benefits, such as food production, shoreline stabilisation, recreation, or storm protection, to people (Díaz et al. 2015; La Notte et al. 2017). The receipt of benefits is moderated by people’s needs, values, access, and sociocultural background (Daw et al. 2016; Lau et al. 2019), but how these societal variables influence benefits is little-researched. Benefits from ecosystems may also change seasonally in ways that are poorly understood and seldom incorporated in management strategies, despite their potential implications for gender equity and household well-being (Grantham et al. 2021). Research on this challenge in the Pacific region will seek to identify emerging concerns and opportunities, their impacts on equity and well-being, and possible societal responses. For example, awareness of a possible future shortfall in small-scale fisheries led to the introduction of the marine snail *Rochia nilotica* to Western Samoa during the period 2003–2006, resulting in the successful creation of a new fishery with substantial benefits and improved livelihood diversification for the local community (Purcell et al. 2021).

Understanding of the costs and benefits resulting from management interventions in seascapes is however still crude and is often divorced from broader decision-making. Key decisions are often made in the absence of publicly available cost-benefits analyses. When these are available, they generally do not account adequately for non-market values (Akter et al. 2014). Tradeoffs between sectors are insufficiently documented, particularly where they link land and sea; and better understandings of the underlying values at stake, and the causes and social and ecological effects of decisions over space and time, are needed to inform policies that create fewer losers. For example, public subsidies for agriculture, mining, and forestry in catchments draining into Australia’s Great Barrier Reef (GBR) increase sediment and pollutant loads, reducing the quality of the natural environment and affecting tourism and fisheries (Brodie and Pearson 2016). An improved understanding of the complex interactions that underpin systemic responses, and how these interactions enhance or reduce desirable system resilience, will be essential for informed comparisons of alternative management strategies (Grafton et al. 2019).

Addressing the third challenge (how science and society can together improve sustainability) requires research that identifies which capacities must be developed and what actions undertaken at different scales, and by whom, to ensure a just and sustainable future for seascapes. We identified two key research priorities: improving seascape technologies and practices (RP5), and learning to govern and manage seascapes for all (RP6; Table 1). Sustainability solutions can arise at a local level — through the actions of individuals, communities, and industries — and at regional, national, and global levels, through the creation and negotiation of norms, laws, policies, and economic systems that incentivise and support responsible and sustainable interactions between people and ecosystems (Charles 2012; Orach et al. 2017). Research that identifies and tests novel, sustainable interventions that help populations to adapt while continuing to enjoy key needs and values is increasingly necessary. At present, for example, sea-walls are the dominant response to erosion on coral coasts, while nature-based solutions that may entail far fewer costs and risks remain neglected due to a lack of knowledge about their technical and social feasibility. Implementation of such solutions requires intensive, coordinated engagement with local communities and real-time, high-resolution monitoring, mapping, and modelling to identify biophysical causes of seascape changes and their solutions (Hickey et al. 2020). Research that develops observing tools and technologies, information systems, analysis, and forecasts can be further supported by coordination and leadership from intergovernmental initiatives such as the Global Ocean Observing System (GOOS; https://www.goosocean.org/).

Addressing uncertainty about changes and interactions, as well as about effective and fair responses, requires knowledge creation from diverse sources to be integrated throughout seascape governance processes so that research can iteratively learn from, respond to, and inform governance (and vice versa). Policymakers, managers, and communities continue to struggle with designing institutions and making decisions that reflect the environmental complexity and human diversity of seascapes. Government departments are often siloed; cumulative assessment, strategic and adaptive planning tools are not always implemented well (Foley et al. 2017); and novel solutions are undermined when influential actors are not engaged in decision-making (Turner et al. 2016). Integration across the relevant communities of researchers, policy makers, managers, and residents should ideally follow procedural, professional, evaluative, judicial, instrumental, and external controls that support scientific and decision-making integrity by individuals and agencies (Colloff et al. 2021). The question of how to identify and assist keystone actors to modify their behaviours (e.g. through diplomacy, supply chains, diffusion of information and technology, and/or niche behaviours), and embed them in stable routines and practices, is a critical research frontier that is essential to navigating emerging seascape conflicts and avoiding potentially maladaptive interventions (Abbott et al. 2016; Österblom et al. 2017). Similarly, rapidly developing bioprospecting and energy proposals (energy, minerals, chemicals, and pharmaceuticals) and adaptive interventions (restoration, geo-engineering, and bioengineering) in tropical seascapes are raising important questions about governance, power, ethics, ownership, and equity that require urgent resolution (Blasiak et al. 2018).

Research is also needed to understand how change in governance systems is influenced by entrenched power asymmetries within and across national, regional, and global levels; by similarly path-dependent systems of economic production and distribution; by systemic risks; and by assumptions about change and resilience (Cumming and Peterson 2017; Morrison et al. 2019). These influences on governance are further modulated by ‘bad faith’ actors (e.g. lobby campaigns, groups with vested interests in particular sectors), disinformation, and self-interest. By evaluating alternatives and projecting outcomes, and by engaging directly with key actors, research can support SDG 14 by spanning the gap between powerful actors in the mainstream and weak and marginal actors in the margins to help both sets of actors identify new and alternative pathways to desirable futures (Tomich et al. 1998).

In addition to priorities that fall mainly under individual challenges, we identified three cross-cutting priorities that address all three challenges: sustainable use, justice, and human well-being (RP7); bridging communities and epistemologies (world views) for innovative, equitable, and scale-crossing solutions (RP8); and informing resilient seascape futures, primarily by using models and related methods (e.g. scenario planning) to understand seascape dynamics and provide decision support (RP9). As explained in Table 1, RP7 and RP8 are integral to the process of transdisciplinary research in each of the other priority areas and understanding their role in sustainability will be critical for achieving SDG14. The use of a diverse range of models and methods (RP9), across a gradient from the fully quantitative through to primarily qualitative, will be vital for explaining and developing approaches for testing and refining hypotheses about interactions, feedbacks, and other complex phenomena that occur over broad extents and long time periods (Lenton 2020).

The experience of well-being and its dependence on seascapes varies significantly across individuals and societies. Further research is needed to understand what constitutes well-being; how it changes over time; and how different aspects of human well-being and anthropogenic impacts on ecosystems are inter-related. Both exposure to disturbance and human response capacity are intricately connected to access to resources (Finkbeiner et al. 2018), which at a community level is fundamentally a question of equity and justice. Thus, research on how equity influences decision-making is fundamental to understanding societal responses to ecological change. An increasing amount of research is showing that research process is critical for successful outcomes. Knowledge co-production research and practice hold potential for innovative, equitable, and effective sustainability solutions (Chambers et al. 2021; Zurba et al. 2021), but many unanswered questions remain about how to most effectively engage diverse stakeholders, recognise unique knowledge (Aminpour et al. 2021; Zurba et al. 2021), and build adaptable knowledge networks, institutional support, and resourcing to ensure a meaningful and Indigenous community-led translation of research into action (Austin et al. 2019; Thornton and Scheer 2012). Gaps remain between high-level commitments and policy and practice that meaningfully bridge diverse epistemologies (‘ways of knowing’) (Witter and Satterfield 2019), suggesting a lack of attention to the roles of power and politics in co-production outcomes, including failures (Turnhout et al. 2020). Meaningful co-production remains nascent in marine science (Hedge et al. 2020) and ocean commons (Vierros et al. 2020).

The interdisciplinary, social-ecological science needed to proactively build sustainable seascapes requires an interplay of empirical data, models, and theory that connect specific systems and locations at different scales and different levels of generality (Cumming et al. 2020, 2017; Meyfroidt et al. 2018). Ecosystem models are widely used to support management decisions; for example, data for larval dispersal (Paris et al. 2013) have been extended to understand the fisheries benefits of Marine Protected Areas from larval spill-over (Krueck et al. 2017), but these models do not generally incorporate human decision-making.

The contributions of explicitly social-ecological models to seascape sustainability range from stringent but highly specific prediction through to simpler but very general conceptual aids and tests of heuristics. Kasperski et al. (2021) grouped social-ecological fisheries models into seven categories: end-to-end models, conceptual models, bioeconomic models, management strategy evaluations, fisher behaviour models, integrated social vulnerability models, and regional economic impact models. Other models of relevant social and socioeconomic processes include (for example) models of the influence of learning on the effectiveness of different management styles (Lindkvist and Norberg 2014), and decision-making in multi-agent systems (Müller et al. 2013). However, linking biophysical ecosystem functions and social drivers of human behaviour to one another, and to social outcomes, remains a significant research challenge.

**Fig. 3:**
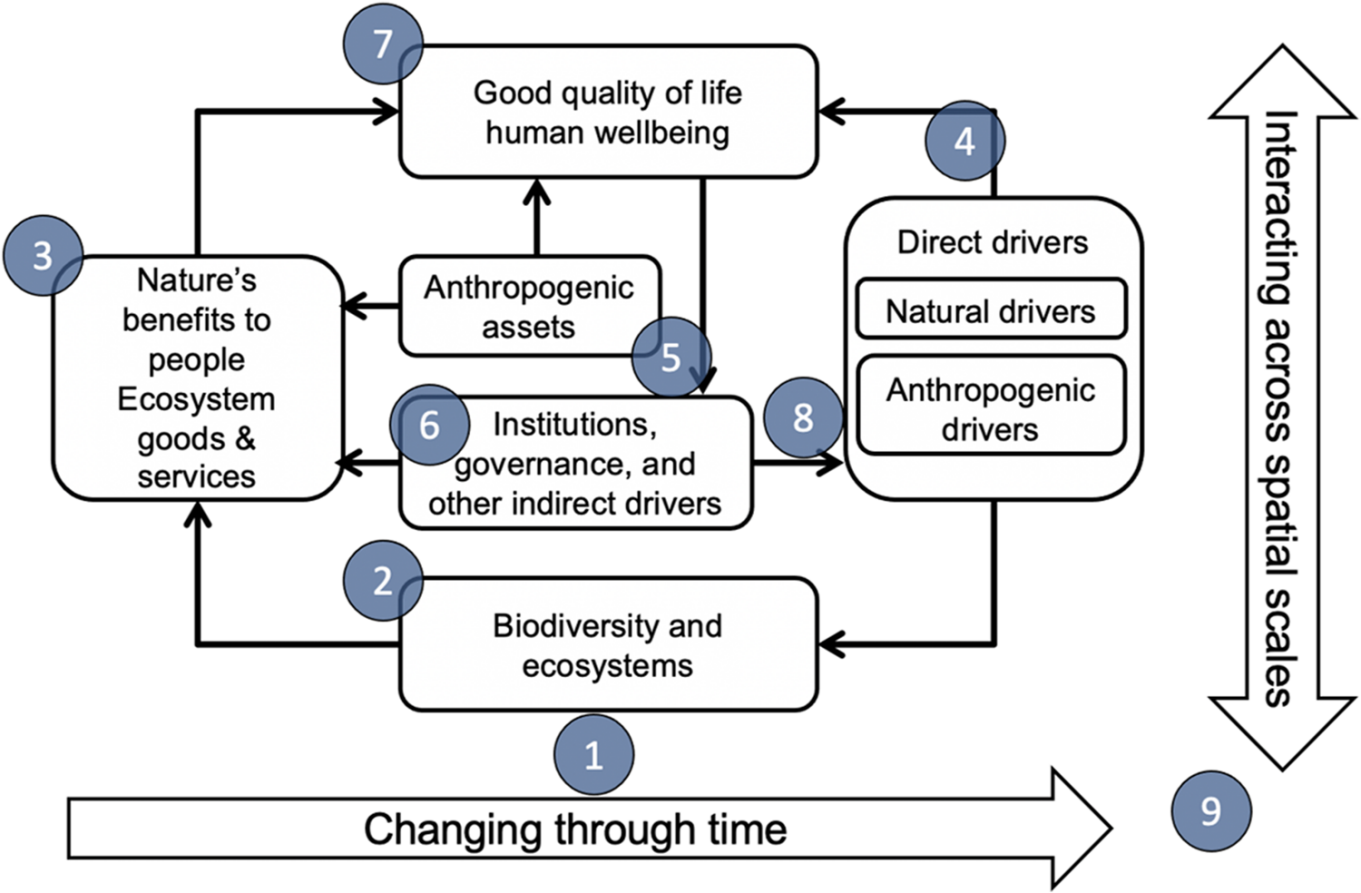
Relationship of each of the nine research priorities to the International Panel on Biodiversity and Ecosystem Services (IPBES) conceptual framework. Modified from Díaz et al. (2015)and a recent summary of knowledge gaps (Mastrángelo et al. 2019). Numbers correspond to research priority numbers in Table 1; arrows highlighting the importance of space and scale are not intended as axes

Taken as a group, there remain many questions about how these research priorities could be addressed in practice, in part because they are context dependent. We regard identification of shared research questions as a critical step towards understanding how existing research initiatives can contribute to the SDGs, where priority areas fall for future research, and where diverse teams and disciplines can work together. Our research priorities for the future contribute key insights in support of the holistic vision of the UN SDGs (Lynch et al. 2020), including addressing whole of system knowledge gaps critical to the goals of Sustainable Cities and Communities (SDG 11), Climate Action (SDG 13), and Life Below Water (SDG 14). They also relate closely to other global initiatives and assessments. This is not an appropriate place for a full listing of such initiatives, but some well-known examples with high relevance for tropical seascapes include the International Panel on Biodiversity and Ecosystem Services (IPBES, Fig. 3), the Program on Ecosystem Change and Society (PECS), and the Coral Triangle Initiative (CTI). IPBES seeks to generate information to monitor and promote both biodiversity conservation and sustainable use of ecosystem services; PECS shares the same goal, but through a more place-focused approach; and the CTI was intended to stimulate improved management of natural resources for sustainable livelihoods across one of the ocean’s most biodiverse regions, the Coral Triangle. Our findings can help these initiatives to understand and frame how research can contribute more effectively to a desirable future. We acknowledge that identifying pathways to impact requires a much larger and more inclusive exercise that is beyond the scope of this synthesis.

## Discussion and conclusions

While a concerted global effort to sustain life below water (SDG 14) is urgently needed, many nations have limited power and capacity to respond effectively to current environmental trends (Barlow et al. 2018; Morrison et al. 2020). By highlighting issues of environmental-social justice and equity and the critical role that they play in seascape sustainability, and engaging deeply with partners on the ground, researchers have the potential to create new ideas, networks, practices, norms, and solutions for the sustainability of tropical seascapes. Solving emerging problems in seascape conservation and management will require intensive learning processes that generate new knowledge and partnerships across a wide diversity of knowledge systems, and that build effective processes for transforming knowledge into action (Clark et al. 2016). For example, Marine Protected Areas (MPAs) have long been considered a critical element of effective seascape conservation, with a considerable body of research demonstrating how MPAs can support both conservation and livelihood objectives of SDG 14 (Di Lorenzo et al. 2020; Edgar et al. 2014; Fox et al. 2014). With increasing criticism by stakeholders and researchers of decisions about MPA location, creation, and management (Hogg et al. 2017; O’Leary et al. 2018), research has also contributed usefully to innovative co-management solutions that benefit both people and ecosystems (Smallhorn‐West et al. 2020).

We have described nine inter-related research priorities that we regard as central to ensuring the future sustainability of seascapes around the world. These priorities are linked by many important cross-cutting research themes. For example, understanding the interplay of local and regional influences on ecological communities, and the feedbacks between local and regional social, ecological, and social-ecological dynamics, will be critical for efforts to build local resilience to ongoing threats such as anthropogenic climate change, over-fishing, and pollution (Hughes et al. 2017a). Similarly, changes in human wealth and lifestyles, and related demands for marine resources, will affect not only patterns of marine resource consumption and pollution but also the nature of ecosystem service demands placed on marine ecosystems and the ways in which societies evaluate and resolve the tradeoffs between different ecosystem services (Crona et al. 2020; Lapointe et al. 2019). Against a backdrop of increasing urbanisation and coastal settlement, research focusing on SDG 14 must anticipate future knowledge needs and the potential for rapid changes in marine access and resource requirements as well as the creation of new forms of social injustice (Bennett et al. 2021).

Research agendas are created by people who are embedded within particular cultures and social strata, including both researchers and funders, and the questions that individuals and groups consider to be important or interesting strongly reflect their own values, background, and preferences (Watkins and Harvey 2020); we do not expect to be immune from this effect. The only proven route to overcoming bias in science — and to achieving broader impact — is to work in diverse, multi-cultural teams and engage strongly with other members of society (i.e. outside the research group) during the research process (Smith-Doerr et al. 2017). Thus, we strongly support critical discussion of this framework and the arguments made by others for transdisciplinary marine and coastal science that engages in co-design and co-production between scientists and society throughout the process of identifying problems, formulating and developing questions, research design, analysis, communication, and translation to action (Chambers et al. 2021; Nakata 2002).

There is clearly no single panacea for marine and coastal sustainability, and making better progress towards SDG 14 in the western Pacific will require a complex and challenging series of interventions. Environmental degradation and rising inequality around the world suggest limited success for standard research approaches promoting science as a tool to solve environmental problems. We nonetheless believe that research has critical roles to play in the generation of new knowledge, the expansion and sharing of grassroots innovations (Seyfang and Smith 2007), and the engagement of broader society to co-develop a nuanced, evidence-based understanding of seascapes and their futures. Most of the underlying social, economic, and ecological problems currently faced by seascapes cannot be resolved by the same thinking that created them. Cross-cutting, transdisciplinary research can generate missing knowledge about complex social-ecological dynamics while helping to identify locally feasible solutions and supporting cross-scale actions to build resilience in both human and ecological communities (Cumming et al. 2017; Folke et al. 2016).

## Data Availability

All data are available in the main text.

## References

[CR1] Abbott KW, Green JF, Keohane RO (2016). Organizational ecology and institutional change in global governance. Int Org.

[CR2] Akter S, Grafton RQ, Merritt WS (2014). Integrated hydro-ecological and economic modeling of environmental flows: Macquarie Marshes, Australia. Agric Wat Manage.

[CR3] Aminpour P, Gray SA, Singer A, Scyphers SB, Jetter AJ (2021). The diversity bonus in pooling local knowledge about complex problems. Proc Nat Acad Sci USA.

[CR4] Austin B, Robinson C, Mathews D, Oades D, Wiggin A (2019). An Indigenous-led approach for regional knowledge partnerships in the Kimberley region of Australia. Hum Ecol.

[CR5] Barlow J, França F, Gardner TA, Hicks CC, Lennox GD (2018). The future of hyperdiverse tropical ecosystems. Nature.

[CR6] Barnes ML, Wang P, Cinner JE, Graham NA, Guerrero AM (2020). Social determinants of adaptive and transformative responses to climate change. Nat Clim Change.

[CR7] Bellwood DR, Streit RP, Brandl SJ, Tebbett SB (2019). The meaning of the term ‘function’in ecology: a coral reef perspective. Fun Ecol.

[CR8] Bennett NJ, Blythe J, White CS, Campero C (2021). Blue growth and blue justice: ten risks and solutions for the ocean economy. Mar Policy.

[CR9] Bernard A, Rodrigues AS, Cazalis V, Grémillet D (2021). Toward a global strategy for seabird tracking. Cons Let.

[CR10] Blasiak R, Jouffray J-B, Wabnitz CC, Sundström E, Österblom H (2018). Corporate control and global governance of marine genetic resources. Sci Adv.

[CR11] Brodie J, Pearson RG (2016). Ecosystem health of the Great Barrier Reef: time for effective management action based on evidence. Estuar Coast Shelf Sci.

[CR12] Burge CA, Mark Eakin C, Friedman CS, Froelich B, Hershberger PK (2014). Climate change influences on marine infectious diseases: implications for management and society. Ann Rev Mar Sci.

[CR13] Chambers JM, Wyborn C, Ryan ME, Reid RS, Riechers M (2021). Six modes of co-production for sustainability. Nat Sust.

[CR14] Charles A (2012). People, oceans and scale: governance, livelihoods and climate change adaptation in marine social-ecological systems. Curr Op Env Sust.

[CR15] Cinner JE, Daw T, Huchery C, Thoya P, Wamukota A (2014). Winners and losers in marine conservation: fishers’ displacement and livelihood benefits from marine reserves. Soc & Nat Res.

[CR16] Clark WC, Tomich TP, Van Noordwijk M, Guston D, Catacutan D (2016). Boundary work for sustainable development: natural resource management at the Consultative Group on International Agricultural Research (CGIAR). Proc Nat Acad Sci USA.

[CR17] Coleman M, Wernberg T (2020). The silver lining of extreme events. Trends Ecol Evo.

[CR18] Colloff MJ, Grafton RQ, Williams J (2021). Scientific integrity, public policy and water governance in the Murray-Darling Basin, Australia. Australas J Water Resour.

[CR19] Crona B, Wassénius E, Troell M, Barclay K, Mallory T (2020). China at a crossroads: an analysis of China’s changing seafood production and consumption. One Earth.

[CR20] Cumming GS, Peterson GD (2017). Unifying research on social-ecological resilience and collapse. Trends Ecol Evol.

[CR21] Cumming GS, Alcamo J, Sala O, Swart R, Bennett EM (2005). Are existing global scenarios consistent with ecological feedbacks?. Ecosystems.

[CR22] Cumming GS, Morrison TH, Hughes TP (2017). New directions for understanding the spatial resilience of social-ecological systems. Ecosystems.

[CR23] Cumming G, Epstein G, Anderies J, Apetrei C, Baggio J (2020). Advancing understanding of natural resource governance: a post-Ostrom research agenda. Curr Op Env Sust.

[CR24] Daw T, Hicks C, Brown K, Chaigneau T, Januchowski-Hartley F (2016). Elasticity in ecosystem services: exploring the variable relationship between ecosystems and human well-being. Ecol Soc.

[CR25] Di Lorenzo M, Guidetti P, Di Franco A, Calò A, Claudet J (2020). Assessing spillover from marine protected areas and its drivers: a meta-analytical approach. Fish Fish.

[CR26] Díaz S, Demissew S, Carabias J, Joly C, Lonsdale M (2015). The IPBES Conceptual Framework-connecting nature and people. Curr Op Env Sust.

[CR27] Donelson JM, Sunday JM, Figueira WF, Gaitán-Espitia JD, Hobday AJ (2019). Understanding interactions between plasticity, adaptation and range shifts in response to marine environmental change. Phil Trans Roy Soc B.

[CR28] Duarte CM, Krause-Jensen D (2018). Intervention options to accelerate ecosystem recovery from coastal eutrophication. Front Mar Sci.

[CR29] Dunic JC, Brown CJ, Connolly RM, Turschwell MP, Cote IM (2021). Long-term declines and recovery of meadow area across the world’s seagrass bioregions. Glob Change Biol.

[CR30] Eakin H, DeFries R, Kerr S, Lambin EF, Liu J, Seto KC, Reenberg A (2014). Significance of telecoupling for exploration of land-use change. Rethinking global land use in an urban era.

[CR31] Eddy TD, Lam VW, Reygondeau G, Cisneros-Montemayor AM, Greer K (2021). Global decline in capacity of coral reefs to provide ecosystem services. One Earth.

[CR32] Edgar GJ, Stuart-Smith RD, Willis TJ, Kininmonth S, Baker SC (2014). Global conservation outcomes depend on marine protected areas with five key features. Nature.

[CR33] Engler C (2020) Transboundary fisheries, climate change, and the ecosystem approach: taking stock of the international law and policy seascape. Ecol Soc 25: Artn 4310.5751/ES-11988–250443

[CR34] FAO (2020) The State of world fisheries and aquaculture (SOFIA) 2020 Report. USA, Rome, pp. 244pp.

[CR35] Feeley KJ, Stroud JT, Perez TM (2017). Most ‘global’ reviews of species’ responses to climate change are not truly global. Divers Distrib.

[CR36] Finkbeiner EM, Micheli F, Bennett NJ, Ayers AL, Le Cornu E (2018). Exploring trade-offs in climate change response in the context of Pacific Island fisheries. Mar Policy.

[CR37] Fischer J, Lindenmayer DB, Manning AD (2006). Biodiversity, ecosystem function, and resilience: ten guiding principles for commodity production landscapes. Front Ecol Env.

[CR38] Fisher R, Radford BT, Knowlton N, Brainard RE, Michaelis FB (2011). Global mismatch between research effort and conservation needs of tropical coral reefs. Cons Let.

[CR39] Foley MM, Mease LA, Martone RG, Prahler EE, Morrison TH (2017). The challenges and opportunities in cumulative effects assessment. Env Impact Assess Rev.

[CR40] Folke C, Biggs R, Norström AV, Reyers B, Rockström J (2016). Social-ecological resilience and biosphere-based sustainability science. Ecol Soc.

[CR41] Fox HE, Holtzman JL, Haisfield KM, McNally CG, Cid GA (2014). How are our MPAs doing? Challenges in assessing global patterns in marine protected area performance. Coast Manage.

[CR42] Friess DA, Rogers K, Lovelock CE, Krauss KW, Hamilton SE (2019). The state of the world’s mangrove forests: past, present, and future. Ann Rev Env Res.

[CR43] Gibson K, Haslam N, Kaplan I (2019). Distressing encounters in the context of climate change: idioms of distress, determinants, and responses to distress in Tuvalu. Transcult Psych.

[CR44] Gormley KS, Hull AD, Porter JS, Bell MC, Sanderson WG (2015). Adaptive management, international co-operation and planning for marine conservation hotspots in a changing climate. Mar Policy.

[CR45] Grafton RQ, Doyen L, Béné C, Borgomeo E, Brooks K (2019). Realizing resilience for decision-making. Nat Sust.

[CR46] Graham NA, Wilson SK, Carr P, Hoey AS, Jennings S (2018). Seabirds enhance coral reef productivity and functioning in the absence of invasive rats. Nature.

[CR47] Grantham R, Álvarez-Romero JG, Mills DJ, Rojas C, Cumming GS (2021). Spatiotemporal determinants of seasonal gleaning. Peop Nat.

[CR48] Hedge P, Van Putten EI, Hunter C, Fischer M (2020). Perceptions, motivations and practices for Indigenous engagement in marine science in Australia. Front Mar Sci.

[CR49] Hickey SM, Radford B, Roelfsema CM, Joyce KE, Wilson SK (2020). Between a reef and a hard place: capacity to map the next coral reef catastrophe. Front Mar Sci.

[CR50] Hill R, Bellgrove A, Macreadie PI, Petrou K, Beardall J (2015). Can macroalgae contribute to blue carbon? An Australian perspective. Limnol Oceanog.

[CR51] Hodgson EE, Halpern BS, Essington TE (2019). Moving beyond silos in cumulative effects assessment. Front Ecol and Evol.

[CR52] Hogg K, Noguera-Méndez P, Semitiel-García M, Gray T, Young S (2017). Controversies over stakeholder participation in marine protected area (MPA) management: a case study of the Cabo de Palos-Islas Hormigas MPA. Ocean Coast Manage.

[CR53] Hughes TP, Bellwood DR, Folke C, Steneck RS, Wilson J (2005). New paradigms for supporting the resilience of marine ecosystems. Trends Ecol Evol.

[CR54] Hughes TP, Barnes ML, Bellwood DR, Cinner JE, Cumming GS (2017). Coral reefs in the Anthropocene. Nature.

[CR55] Hughes TP, Kerry JT, Álvarez-Noriega M, Álvarez-Romero JG, Anderson KD (2017). Global warming and recurrent mass bleaching of corals. Nature.

[CR56] Jouffray J-B, Blasiak R, Norström AV, Österblom H, Nyström M (2020). The blue acceleration: the trajectory of human expansion into the ocean. One Earth.

[CR57] Karki M, Sellamuttu SS, Okayasu S, Suzuki W, Acosta LA (2018). IPBES Summary for policymakers of the regional assessment report on biodiversity and ecosystem services for Asia and the Pacific of the Intergovernmental Science-Policy Platform on Biodiversity and Ecosystem Services.

[CR58] Kasperski S, DePiper GS, Haynie AC, Blake S, Colburn LL (2021). Assessing the state of coupled social-ecological modeling in support of ecosystem based fisheries management in the United States. Front Mar Sci.

[CR59] Kelly C, Ellis G, Flannery W (2019). Unravelling persistent problems to transformative marine governance. Front Mar Sci.

[CR60] Könnölä T, Salo A, Cagnin C, Carabias V, Vilkkumaa E (2012). Facing the future: scanning, synthesizing and sense-making in horizon scanning. Sci Public Policy.

[CR61] Krueck N, Ahmadia GN, Green A, Jones GP, Possingham HP (2017). Incorporating larval dispersal into MPA design for both conservation and fisheries. Ecol App.

[CR62] La Notte A, D’Amato D, Mäkinen H, Paracchini ML, Liquete C (2017). Ecosystem services classification: a systems ecology perspective of the cascade framework. Ecol Indic.

[CR63] Lam VW, Allison EH, Bell JD, Blythe J, Cheung WW (2020). Climate change, tropical fisheries and prospects for sustainable development. Nat Rev Earth Env.

[CR64] Lapointe M, Cumming GS, Gurney GG (2019). Comparing ecosystem service preferences between urban and rural dwellers. Bioscience.

[CR65] Lapointe M, Gurney GG, Cumming GS (2020). Urbanization alters ecosystem service preferences in a Small Island Developing State. Eco Serv.

[CR66] Lau JD, Hicks CC, Gurney GG, Cinner JE (2019). What matters to whom and why? Understanding the importance of coastal ecosystem services in developing coastal communities. Eco Serv.

[CR67] Lenton TM (2020). Tipping positive change. Phil Trans Roy Soc B.

[CR68] Lindkvist E, Norberg J (2014). Modeling experiential learning: the challenges posed by threshold dynamics for sustainable renewable resource management. Ecol Econ.

[CR69] Liu J, Dietz T, Carpenter SR, Alberti M, Folke C (2007). Complexity of coupled human and natural systems. Science.

[CR70] Liu J, Mooney H, Hull V, Davis SJ, Gaskell J, Hertel T, Lubchenco J, Seto KC, Gleick P, Kremen C, Li S (2015) Systems integration for global sustainability. Science 347(6225):1258832. 10.1126/science.1258832 10.1126/science.1258832 25722418

[CR71] Lynch AJ, Elliott V, Phang SC, Claussen JE, Harrison I (2020). Inland fish and fisheries integral to achieving the Sustainable Development Goals. Nat Sust.

[CR72] Mastrángelo ME, Pérez-Harguindeguy N, Enrico L, Bennett E, Lavorel S (2019). Key knowledge gaps to achieve global sustainability goals. Nat Sust.

[CR73] Mellin C, Hicks CC, Fordham DA, Golden CD, Kjellevold M (2022). Safeguarding nutrients from coral reefs under climate change. Nat Eco Evo.

[CR74] Menegotto A, Rangel TF (2018). Mapping knowledge gaps in marine diversity reveals a latitudinal gradient of missing species richness. Nat Comms.

[CR75] Meyfroidt P, Chowdhury RR, de Bremond A, Ellis EC, Erb K-H (2018). Middle-range theories of land system change. Global Env Change.

[CR76] Morais RA, Bellwood DR (2019). Pelagic subsidies underpin fish productivity on a degraded coral reef. Curr Biol.

[CR77] Morais RA, Depczynski M, Fulton C, Marnane M, Narvaez P (2020). Severe coral loss shifts energetic dynamics on a coral reef. Fun Ecol.

[CR78] Morrison T, Adger WN, Brown K, Lemos MC, Huitema D (2019). The black box of power in polycentric environmental governance. Glob Env Change.

[CR79] Morrison TH, Adger N, Barnett J, Brown K, Possingham H (2020). Advancing coral reef governance into the Anthropocene. One Earth.

[CR80] Murray NJ, Phinn SR, DeWitt M, Ferrari R, Johnston R (2019). The global distribution and trajectory of tidal flats. Nature.

[CR81] Nakata M (2002). Indigenous knowledge and the cultural interface: underlying issues at the intersection of knowledge and information systems. IFLA J.

[CR82] Nickols KJ, White JW, Malone D, Carr MH, Starr RM (2019). Setting ecological expectations for adaptive management of marine protected areas. J Appl Ecol.

[CR83] Nunan F, Omond MA, Nchimbi AY, Mangora MM, Kairoe JG (2020). The silos of natural resource governance. Cons Soc.

[CR84] O’Leary BC, Ban NC, Fernandez M, Friedlander AM, García-Borboroglu P (2018). Addressing criticisms of large-scale marine protected areas. Bioscience.

[CR85] Opdam P, Luque S, Nassauer J, Verburg PH, Wu J (2018). How can landscape ecology contribute to sustainability science?. Land Ecol.

[CR86] Orach K, Schlüter M, Österblom H (2017). Tracing a pathway to success: how competing interest groups influenced the 2013 EU Common Fisheries Policy reform. Env Sci Pol.

[CR87] Österblom H, Jouffray J-B, Folke C, Rockström J (2017). Emergence of a global science-business initiative for ocean stewardship. Proc Nat Acad Sci USA.

[CR88] Oziolor EM, Reid NM, Yair S, Lee KM, VerPloeg SG (2019). Adaptive introgression enables evolutionary rescue from extreme environmental pollution. Science.

[CR89] Partelow S, Schlüter A, von Wehrden H, Jänig M, Senff P (2018). A sustainability agenda for tropical marine science. Cons Let.

[CR90] Pittman SJ (2017). Seascape Ecology.

[CR91] Pittman S, Yates K, Bouchet P, Alvarez-Berastegui D, Andréfouët S (2021). Seascape ecology: identifying research priorities for an emerging ocean sustainability science. Mar Ecol Prog Ser.

[CR92] Poiani KA, Richter BD, Anderson MG, Richter HE (2000). Biodiversity conservation at multiple scales: functional sites, landscapes, and networks. Bioscience.

[CR93] Pomeroy R, Garces L, Pido M, Silvestre G (2010). Ecosystem-based fisheries management in small-scale tropical marine fisheries: emerging models of governance arrangements in the Philippines. Mar Policy.

[CR94] Purcell S, Tagliafico A, Cullis B, Gogel B (2021). Socioeconomic impacts of resource diversification from small-scale fishery development. Ecol Soc.

[CR95] Robinson JP, Wilson SK, Robinson J, Gerry C, Lucas J (2019). Productive instability of coral reef fisheries after climate-driven regime shifts. Nat Ecol Evol.

[CR96] Rocha JC, Peterson G, Bodin Ö, Levin S (2018). Cascading regime shifts within and across scales. Science.

[CR97] Said A, Chuenpagdee R (2019). Aligning the sustainable development goals to the small-scale fisheries guidelines: a case for EU fisheries governance. Mar Policy.

[CR98] Sanford E, Kelly MW (2011). Local adaptation in marine invertebrates. Ann Rev Mar Sci.

[CR99] Saunders MI, Leon JX, Callaghan DP, Roelfsema CM, Hamylton S (2014). Interdependency of tropical marine ecosystems in response to climate change. Nature Clim Change.

[CR100] Seyfang G, Smith A (2007). Grassroots innovations for sustainable development: towards a new research and policy agenda. Env Policy.

[CR101] Sing Wong A, Vrontos S, Taylor ML (2022). An assessment of people living by coral reefs over space and time. Glob Change Biol.

[CR102] Smallhorn-West PF, Sheehan J, Malimali Sa, Halafihi T, Bridge TC (2020). Incentivizing co-management for impact: mechanisms driving the successful national expansion of Tonga’s Special Management Area program. Cons Let.

[CR103] Smith-Doerr L, Alegria SN, Sacco T (2017). How diversity matters in the US science and engineering workforce: a critical review considering integration in teams, fields, and organizational contexts. Engag Sci Tech Soc.

[CR104] Song AM, Johnsen JP, Morrison TH (2018). Reconstructing governability: how fisheries are made governable. Fish Fish.

[CR105] Spijkers J, Morrison TH, Blasiak R, Cumming GS, Osborne M (2018). Marine fisheries and future ocean conflict. Fish Fish.

[CR106] Sutherland WJ, Woodroof HJ (2009). The need for environmental horizon scanning. Trends Ecol Evo.

[CR107] Tebbett SB, Morais RA, Goatley CH, Bellwood DR (2021). Collapsing ecosystem functions on an inshore coral reef. J Env Manage.

[CR108] Thornton TF, Scheer AM (2012) Collaborative engagement of local and traditional knowledge and science in marine environments: a review. Ecol Soc 17: 10.5751/ES-04714-170308

[CR109] Tomich TP, van Noordwijk M, Vosti SA, Witcover J (1998). Agricultural development with rainforest conservation: methods for seeking best bet alternatives to slash-and-burn, with applications to Brazil and Indonesia. Agric Econ.

[CR110] Turner MG, Gardner RH, O’Neill RV (2001). Landscape ecology in theory and practice: pattern and process. Springer-Verlag.

[CR111] Turner RA, Addison J, Arias A, Bergseth BJ, Marshall NA (2016). Trust, confidence, and equity affect the legitimacy of natural resource governance. Ecol Soc.

[CR112] Turnhout E, Metze T, Wyborn C, Klenk N, Louder E (2020). The politics of co-production: participation, power, and transformation. Curr Op Env Sust.

[CR113] Vierros MK, Harrison A-L, Sloat MR, Crespo GO, Moore JW (2020). Considering Indigenous peoples and local communities in governance of the global ocean commons. Mar Policy.

[CR114] Walsh B, Blows MW (2009). Abundant genetic variation+ strong selection= multivariate genetic constraints: a geometric view of adaptation. Ann Rev Ecol Evol Syst.

[CR115] Warr P (2012) How to think about and measure psychological well-being. In: M. Wang RRSaLET (ed), Research methods in occupational health psychology. Routledge, New York, pp. 100-114. 10.4324/9780203095249-16

[CR116] Watkins TR, Harvey LA (2020). I am not biased. It is everyone else’s problem.

[CR117] Wilson SK, Fulton CJ, Graham NA, AAbesamis R, Berkström C (2022). The contribution of macroalgae-associated fishes to small-scale tropical reef fisheries. Fish Fish.

[CR118] Witter R, Satterfield T (2019). The ebb and flow of Indigenous rights recognitions in conservation policy. Dev Change.

[CR119] Zurba M, Petriello MA, Madge C, McCarney P, Bishop B (2021). Learning from knowledge co-production research and practice in the twenty-first century: global lessons and what they mean for collaborative research in Nunatsiavut. Sust Sci.

